# Smac127 Has Proapoptotic and Anti-Inflammatory Effects on Rheumatoid Arthritis Fibroblast-Like Synoviocytes

**DOI:** 10.1155/2016/6905678

**Published:** 2016-02-17

**Authors:** D. Lattuada, R. Gualtierotti, K. Crotta, P. Seneci, F. Ingegnoli, C. Corradini, R. Viganò, O. Marelli, C. Casnici

**Affiliations:** ^1^Department of Medical Biotechnology and Translational Medicine, School of Medicine, University of Milan, 20129 Milan, Italy; ^2^Division of Rheumatology, A.O. Institute of Gaetano Pini, Department of Clinical Sciences & Community Health, University of Milan, 20122 Milan, Italy; ^3^Department of Chemistry, University of Milan, 20129 Milan, Italy; ^4^Department of Biomedical, Surgical and Dental Sciences, University of Milan c/o Ist Division of Orthopaedy and Traumatology, A.O. Institute of Gaetano Pini, 20112 Milan, Italy; ^5^Department of Physiatry and Rheumatology, Chief of Centre for Rheumatoid Arthritis, A.O. Institute Gaetano Pini, 20122 Milan, Italy; ^6^NEWRONIKA s.r.l., 20122 Milan, Italy; ^7^Fondazione Fernando Santarelli, Systemic Inflammation Lab, 20122 Milan, Italy

## Abstract

Rheumatoid arthritis (RA) is characterized by synovial inflammation and hyperplasia. Fibroblast-like synoviocytes (FLSs) are apoptosis-resistant and contribute to the pathogenesis of RA by producing cytokines and proteolytic enzymes, which degrade the extracellular matrix. We evaluated the proapoptotic and anti-inflammatory activity of the small molecule Smac127 on RA-FLSs cultured in synovial fluid (SF), in order to reproduce the physiopathological environmental characteristic of RA joints. In this context, Smac127 induces apoptosis by inhibiting apoptosis proteins (IAPs). This inhibition activates caspase 3 and restores the apoptotic pathway. In addition, Smac127 induces a significant inhibition of the secretion of IL-15 and IL-6, stimulation of pannus formation, and damage of bone and cartilage in RA. Also the secretion of the anti-inflammatory cytokine IL-10 is dramatically increased in the presence of Smac127. The cartilage destruction in RA patients is partly mediated by metalloproteinases; here we show that the MMP-1 production by fibroblasts cultured in SF is significantly antagonized by Smac127. Conversely, this molecule has no significant effects on RANKL and OPG production. Our observations demonstrate that Smac127 has beneficial regulatory effects on inflammatory state of RA-FLSs and suggest a potential use of Smac127 for the control of inflammation and disease progression in RA.

## 1. Introduction

**Figure 1:**
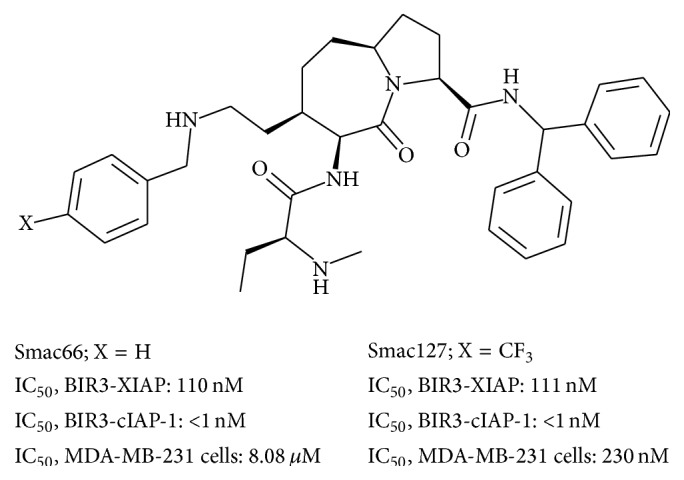
Smac066 and Smac127: chemical structures, cell-free affinity for IAPs, and cytotoxicity against MDA-MB-231 cells.

Rheumatoid arthritis (RA) is characterized by synovial inflammation and hyperplasia, autoantibodies production (rheumatoid factor and anti-citrullinated protein antibodies [ACPA]), and cartilage and bone destruction. Numerous lines of evidence support the potential contribution of fibroblast-like synoviocytes (FLSs) to the pathogenesis of chronic arthritis [1, 2]. In the joints, RA-FLSs display a constitutive proinflammatory phenotype that also persists in tissue culture in the absence of exogenous stimuli [3–5]. This is a tumour-like phenotype that transforms FLSs from fairly “innocent” mesenchymal cells to destructive aggressors, characterized by a number of unique and remarkable features, such as expression of adhesion molecules and mediators, contributing directly to local cartilage destruction and to the chronicity of synovial inflammation [6, 7]. Resistance to apoptosis has also been associated with this specific phenotype of RA-FLSs [8], there is an imbalance between cell death and survival in RA-FLS [9], and the microenvironment also contains, besides the proinflammatory cytokines, factors contributing to induce resistance to apoptosis [10]. It has been suggested that a reduced rate of programmed cell death may provide an explanation for synovial hyperplasia and contribute to invasiveness of RA-FLSs. In the apoptosis resistance are involved the inhibitors of apoptosis proteins (IAPs), a diverse family of proteins that have been implicated as regulators of apoptosis, mitosis, and inflammation [11–13]. However, to date, the overwhelming majority of studies have focused on the ability of these proteins to modulate apoptosis, particularly in the context of TNF receptor engagement, and few studies have explored the role of the IAPs in the production of proinflammatory mediators. Because TNF is a major driver of inflammation in response to infection, as well as in the context of inflammatory diseases [14, 15], the role of IAPs in shaping TNF-dependent inflammatory signalling is an important unresolved question. Recent evidence indicates that cIAP-1 and cIAP-2 play particularly influential roles in repressing TNFR-induced cell death signals. These proteins are characterized by the presence of one to three baculoviral IAP repeat (BIR) domains responsible for the antiapoptotic activity of IAPs because they bind to caspases 3, 7, and 9 and prevent assembly of caspases 8 and 10 [16]. Smac, an endogenous proapoptotic protein, upon release from the mitochondria, binds and antagonizes several members of the IAP family including XIAP, cIAP-1, and cIAP-2. Numerous synthetic Smac mimetic compounds resemble the Smac N-terminal AVP1 sequence and bind to the BIR3 domain on XIAP and IAPs. These compounds also promote IAP ubiquitination and subsequent degradation [17, 18] and endogenous TNF alpha lowers maximum peak bone mass and inhibits osteoblastic Smac activation through NF-*κ*B. In our previous work, we showed that the Smac mimetic Smac066 (Figure 1; X = H) was able to induce apoptosis in RA-FLSs, proving its activity also in inflammatory diseases. In the present study we evaluated the proapoptotic effect of a more potent analogue, Smac127 (Figure 1; X = CF_3_). While Smac66 and Smac127 showed similar binding potency on BIR3 domains from XIAP and cIAP-1 (Figure 1) and were equally stable in biological media, the trifluoromethyl substituent in Smac127 increased its lipophilicity and facilitated its permeation through cell membranes (indirectly measured by ≈30-fold increase in cytotoxicity for Smac127 versus Smac66 (Figure 1)). Finally, we demonstrated the anti-inflammatory activity of Smac127 on RA-FLSs cultured in tissue medium alone or in the presence of RA synovial fluid.

## 2. Materials and Methods

### 2.1. Smac Mimetic Compound

The synthesis of Smac127 was carried out as described elsewhere [19, 20]. Smac127 was dissolved and diluted in distilled sterile water for in vitro administration.

### 2.2. Synovial Fluid Samples and Fibroblast-Like Synoviocytes

All samples of liquid and/or synovial membrane were taken from the materials considered waste during arthrocentesis or surgery, collected by a single team at Ist Division. Local Ethical Committee of A.O. Gaetano Pini Orthopedic Institute and University of Milan approved whole study (approved on 27 March, 2012). All patients signed informed consent to take part in the study.

### 2.3. Synovial Fluid Samples

SF was directly aspirated from the joints of RA patients; the fluid was collected into heparinized tubes and spun at 1000 ×g for 10 min. The acellular portion of SF was stored at −80°C before use. We used pools composed of 10 RA patients' fluid to reduce the variability in responses between the different fluids. SF pool was used at the final dilution of 1 : 8 in culture medium.

### 2.4. Fibroblast-Like Synoviocytes

Synovial tissue was obtained from patients with RA (*n* = 22) during joint synovectomies. Human synovial tissues were digested with collagenase in Dulbecco's Modified Eagle Medium (DMEM) (Euroclone, Pero, Italy) for 2 hrs at 37°C to isolate synoviocytes. Dissociated cells were then centrifuged at 1000 ×g, suspended in DMEM supplemented with 10% FetalClone 1 serum (FCS) (Thermo Scientific, USA), 2 mM L-glutamine, 100 units/mL of penicillin, and 100 *μ*g/mL streptomycin (Euroclone, Pero, Italy), and plated. After overnight culture, nonadherent cells were removed, and adherent cells were cultivated in DMEM supplemented with 10% FCS. The cultures were kept at 37°C in 5% CO_2_ and the medium was replaced every 3 days. The purity of the cells was tested by flow-cytometric analysis using phycoerythrin-conjugated anti-CD14 (Pharmingen, San Diego, CA, USA), fluorescein isothiocyanate phycoerythrin-conjugated anti-CD3, anti-CD19, and anti-Thy-1 (CD90) monoclonal antibodies (R&D Systems, Minneapolis, MN). A FACS Calibur flow cytometer (488Ex/620Em) (Becton Dickinson, San Jose, CA, USA) was used for the analysis. At passage 3, the cells were morphologically homogeneous and exhibited the appearance of FLS, with typical bipolar configuration under inverse microscopy. Most cells (>98%) expressed the surface marker for fibroblasts (Thy-1) and were negative for the expression of CD3, CD19, and CD14. Synoviocytes from passages 3–8 were used in each experiment.

### 2.5. Apoptosis Assay

We assessed apoptosis in RA-FLSs cultured in tissue medium alone or in SF (1 : 8 dilution in culture medium) for 5 days, in the presence/absence of Smac127 (15 *μ*M) for 24 hrs. Apoptotic cells were detected with the Annexin V-FITC apoptosis detection kit (Abcam, Cambridge, UK), according to the manufacturer's instructions. All samples were analysed with a FACS Calibur flow cytometer. FITC-conjugated Annexin V emission was collected in the FLH-1 channel, and propidium iodide (PI, for detecting necrotic or dead cells) emission was collected in the FLH-3 channel. Data were analysed with Cell Quest software. The percentage of apoptosis was calculated, considering cells in both early (Annexin^+^ PI^−^) and late apoptosis (Annexin^+^ PI^+^).

### 2.6. Western Blots

RA-FLSs were grown in culture medium or in SF (1 : 8 dilution in culture medium) for 5 days. Smac127 (15 *μ*M) was added to the cultures 48 hrs before sacrificing the cells for IAPs detections, while for caspase detection Smac127 was added to the culture in medium alone 6 hrs before the sacrifice and to the culture in medium supplemented with SF 18 hrs before the sacrifice. Staurosporine (20 *μ*M) (Sigma-Aldrich, St. Louis, MO) was used as proapoptotic positive control. Cells were lysed in lysis buffer and protein concentration was measured by the BCA method (Thermo Scientific, USA) according to the manufacturer's instructions. The cell lysates were separated by SDS-PAGE on 4–12% Tris-HCl precast gels for IAPs detection, or by 10% Tris-HCl precast gels for caspase 3 detection (Life Technologies, Carlsbad, CA), and transferred onto nitrocellulose membranes (Life Technologies, Carlsbad, CA). The membranes were blocked for 3 hrs with 5% nonfat dry milk (Lab Scientific) in PBS 0.1% Tween-20 (Sigma-Aldrich, St. Louis, MO) and probed with primary antibody overnight at 4°C. The primary antibodies used were cIAP1 (1 : 600) (R&D Systems, Minneapolis), cIAP2 (1 : 600) (BD Pharmingen, MA, USA) and XIAP (1 : 400) (Cell Signaling Technology, Europe), and anticaspase 3 mouse monoclonal antibody (2 *μ*g/mL) (Enzo life Sciences, USA), whereas *β*-actin (1 : 4000) (Sigma-Aldrich, St. Louis, MO) was used as the loading control. Secondary antibodies were conjugated to horseradish peroxidase (Thermo Scientific, USA) and the gels developed using Western Lightning Plus ECL (PerkinElmer, OH, USA). Densitometry was performed using ImageJ software (National Institutes of Health, Bethesda, USA).

### 2.7. Cytokine and Proliferation Assay

RA-FLSs were cultured in culture medium alone or in the presence of SF (1 : 8 dilution in culture medium) for 5 days and treated with Smac127 24 hrs before the sacrifice; then the cells were fixed and permeabilized. To avoid nonspecific binding, the samples were saturated with LI-COR Odyssey Blocking Buffer for 2 hrs with moderate shaking. The plate was washed four times with washing solution (PBS added with 0.1% Tween-20). RA-FLSs were incubated with 50 *μ*L of several primary mouse monoclonal antibodies (anti-human IL-15 (15 *μ*g/mL), IL-6 (15 *μ*g/mL), and IL-10 (15 *μ*g/mL) (Peprotech, Rocky Hill, NJ, USA)) (anti-MMP1(1 : 40), RANKL (1 : 70), and OPG (1 : 15) (Novus, Italy)). After 2.5 hrs, the plate was washed 5 times with PBS + 0.1% Tween-20 and incubated for 1 hr with goat anti-mouse IRDye*™* 800CW labeled secondary antibodies (1 : 800 dilution; LI-COR) and with CellTag 700CW (1 : 500; LI-COR) for cell number normalization. The negative control was obtained incubating cells with secondary antibody alone.

After 5 washes with PBS + 0.1% Tween-20, the plate was scanned simultaneously at 700 nm and 800 nm using the Odyssey infrared imaging system (LI-COR Bioscience). The fluorescently labelled IRDye 800CW secondary antibody was used for detection of a specific cytokine in the 800 nm channel, and two fluorescent cell stains are used in combination in the 700 nm channel to normalize for well-to-well variations in cell number. CellTag 700 Stain is a near-infrared, fluorescent, nonspecific cell stain that provides accurate normalization to cell number. The stain accumulates in both the nucleus and cytoplasm of permeabilized RA-FLS.

### 2.8. Statistical Analysis

Statistical analysis was performed using Student's test for matched pairs. Differences with a confidence level of >95% were considered statistically significant (*p* < 0.05). SPSS 21 (IBM) program will be used.

## 3. Results

**Figure 2:**
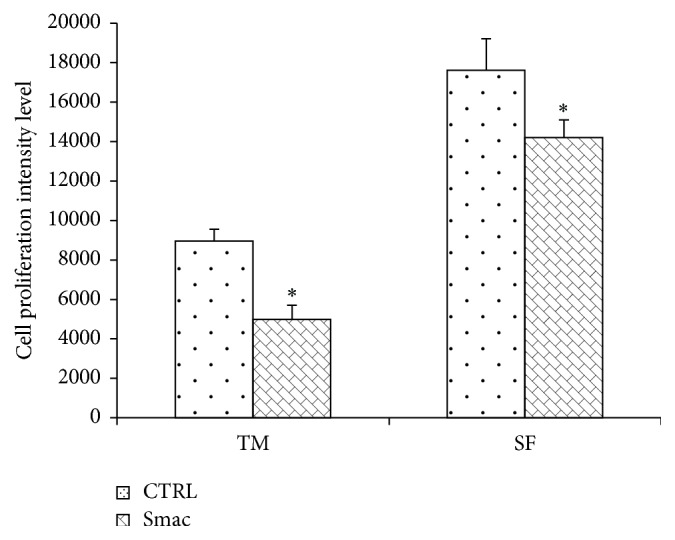
Smac127 inhibits the proliferation in fibroblast-like synoviocytes (FLSs) derived from patients with rheumatoid arthritis (RA). RA-FLSs were cultured in tissue medium alone (TM) or in synovial fluid (SF). CTRL are the cells without Smac127. “Smac” are the cells after the addition of Smac127 (15 *μ*M) for 24 hrs. The data are generated with Odyssey infrared platform. In Cell Western with 700 channels detecting cell proliferation, we detected cytokine production with 800 channels. Results are expressed as the mean percentage ± SD from four individual experiments. ^*∗*^ *p* < 0.05 versus CTRL or SF.

**Figure 3:**
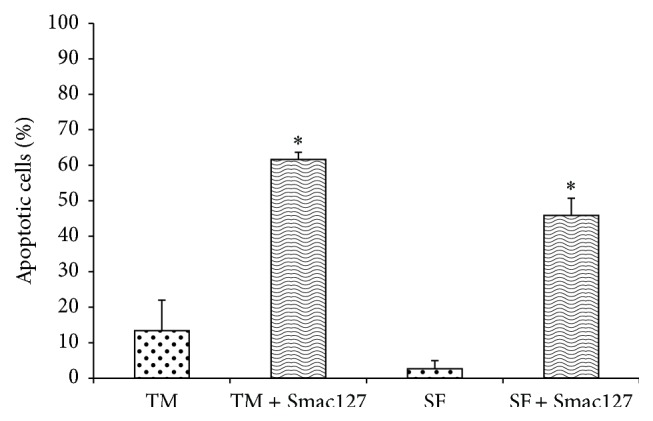
Smac127 induces apoptosis in fibroblast-like synoviocytes (FLSs) derived from patients with rheumatoid arthritis (RA). RA-FLSs were cultured in the presence of tissue medium alone (TM) or in synovial fluid (SF) for 5 days. Smac127 (15 *μ*M) was added for 24 hrs. Apoptosis was detected by Annexin V test. Results are expressed as the mean percentage ± SD from five individual experiments. ^*∗*^ *p* < 0.05 versus CTRL or SF.

In our previous paper we showed that Smac066 induced apoptosis in RA-FLSs [21]; now here we studied Smac127. Smac66 and Smac127 showed similar binding potency on BIR3 domains from XIAP and cIAP-1 (Figure 1) and were equally stable in biological media, but the trifluoromethyl substituent in Smac127 increased its lipophilicity and facilitated its permeation through cell membranes—indirectly measured by ≈30-fold increase in cytotoxicity for Smac127 versus Smac66, which is endowed with greater lipophilicity and intracellular uptake. Since Casnici et al. emphasized the importance of using SF from RA patients in “in vitro” studies involving RA cells, in order to reproduce faithfully the physiopathological environment of RA joints [22], we tested Smac127 on RA-FLSs cultured in SF as well as in tissue medium alone. Our new Smac mimetic compound induced a significant inhibition of cell growth, although lower when cells were cultured in SF (Figure 2). Then, we assessed apoptosis in RA-FLSs cultured in tissue medium alone or in SF, in the presence/absence of Smac127. After 5 days of culture, the addition of Smac127 before the Annexin V test (24 hrs) induced apoptosis and the percentage of apoptotic cells observed in the cultures with SF was lower than those with medium alone but remained significant. Taking into consideration that in the presence of SF a lower percentage of apoptotic cells was initially present than when medium alone was used, probably due to the presence in SF of antiapoptotic factors, the induction of apoptosis by Smac127 was similar in both culture conditions (Figure 3).

**Figure 4:**
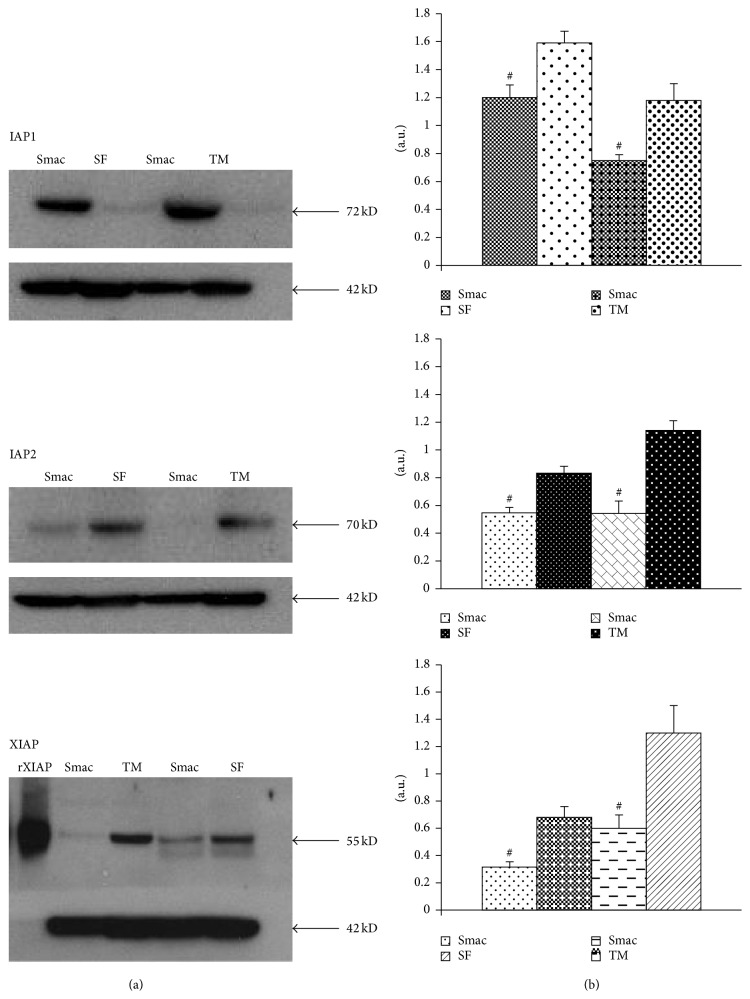
Smac127 inhibits the IAP proteins in RA-FLSs cultured in tissue medium or in synovial fluid. RA-FLSs were grown in tissue medium (TM) or in synovial fluid (SF). Smac127 (15 *μ*M) was added for 48 hrs. (a) Immunoblots show the detection of cIAP1 (72 kDa), cIAP2 (70 kDa), and XIAP (55 kDa). *β*-actin was used as a loading control (42 kDa). (b) Densitometric analyses of the immunoblots show the ratio of IAPs/actin protein expression ± SD of the mean from four independent experiments. ^#^ *p* < 0.05 indicates statistically significant differences compared to untreated RA-FLSs.

**Figure 5:**
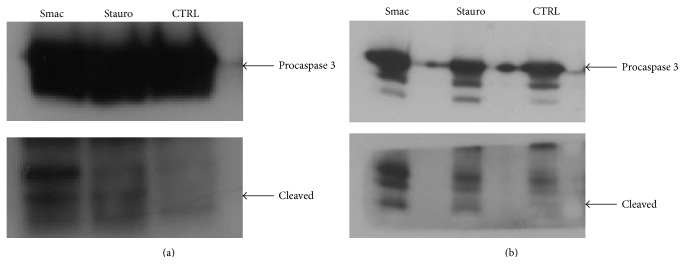
Smac127 induces caspase 3 activation. Western blot analysis shows the effect of Smac127 on caspase 3 in extracts from human fibroblast-like synoviocytes (FLSs) derived from patients with rheumatoid arthritis cultured in TM (a) or in SF for 5 days (b). Levels of cleaved caspase 3 were measured in RA-FLSs untreated (CTRL) or treated with Smac127 (15 *μ*M) or treated with the apoptosis inducer staurosporine (stauro). Cells cultured in tissue medium alone were incubated for 6 hrs, whereas cells grown in the presence of SF were incubated for 18 hrs; staurosporine 20 *μ*M was the positive control.

**Figure 6:**
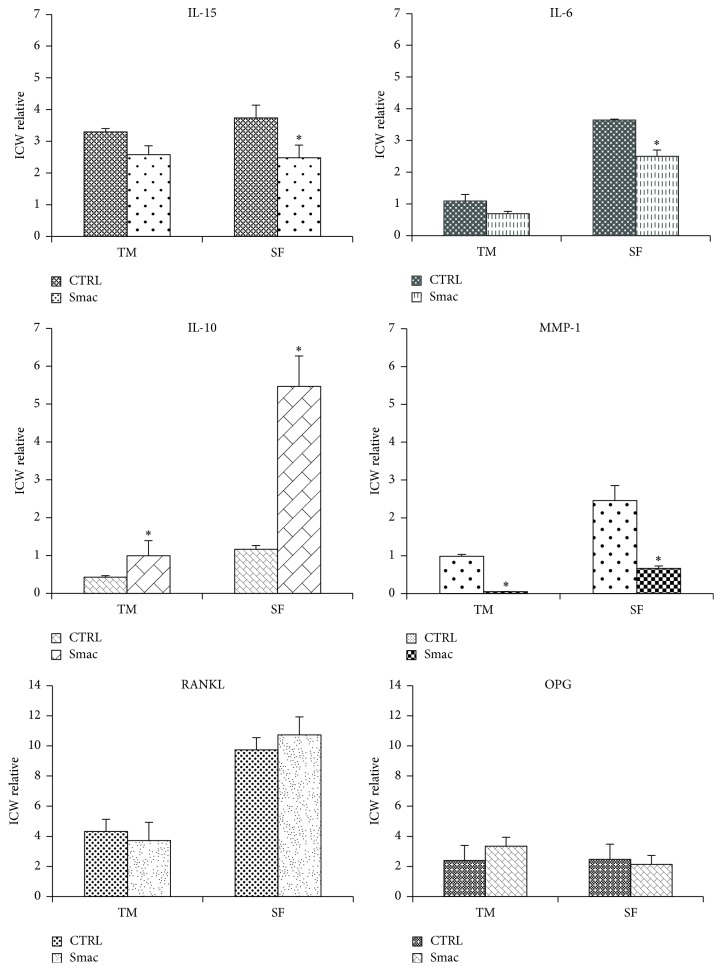
Cytokine modulation in RA-FLSs. IL15: RA-FLSs were grown in tissue medium (TM) or in synovial fluid (SF) for 5 days. Smac127 (15 *μ*M) was added for 24 hrs. The data were generated with Odyssey infrared platform. In Cell Western with 800 channels we detected the cytokine production. Data are expressed as In Cell Western relative data (ICW relative). We analysed the production of IL-15, IL-6, IL-10, MMP-1, RANKL, and OPG. The histograms summarize the mean ± SD of the ten independent experiments. ^*∗*^ *p* < 0.05 versus untreated cells.

Since IAPs are expressed at high levels in RA-FLSs and Smac127 should downregulate these proteins and promote the induction of cell death, we analysed the effect of Smac127 on these proteins. As shown in Figure 4, Smac127 downregulated cIAP1, cIAP2, and XIAP expression in RA-FLSs cultured in tissue medium alone or in the presence of SF. As reported in Figure 5, Smac127 could also promote both the proteolytic activation of procaspase 3 and the enzymatic activity of mature caspase 3. As a positive control, RA-FLSs were incubated with the apoptosis inducer staurosporine. Furthermore, in RA joints, an imbalance between pro- and anti-inflammatory cytokine activities favours the induction of autoimmunity, of chronic inflammation, and thereby of joint damage [15]; therefore, we studied the effects of Smac127 on the modulation of the cytokines expression. In particular, we analysed the production of inflammatory cytokines IL-15 and IL-6 and that of the anti-inflammatory cytokine IL-10. All of these cytokines are known to be involved in RA pathology. As reported in Figure 6, Smac127 induced a significant inhibition on the secretion of IL-15 and IL-6 while the production of anti-inflammatory IL-10 dramatically increased, both in cells cultured in tissue medium alone and in presence of SF (Figure 6). Immune cells and FLSs are the main source of receptor activator of nuclear factor kappa-B ligand (RANKL) in pathological conditions such as arthritic RA joints. The cartilage destruction in RA patients is partly mediated by metalloproteinases secreted by activated synoviocytes and chondrocytes [23]. For this reason we also explored the influence of Smac127 on the RA-FLSs production of metalloprotease 1 (MMP-1), RANKL, and osteoprotegerin (OPG), involved in bone resorption characteristic of RA pathology (Figure 6). The maximum production of MMP-1 occurred when the fibroblasts were cultured for 5 days in SF, and this production was significantly antagonized by Smac127 (Figure 6). Conversely, Smac127 treatment did not influence the secretion of RANKL and OPG.

## 4. Discussion

IAPs are overexpressed in RA-FLSs and contribute to their expansion, inflammation, and disease progression [24] and small molecules that disrupt the binding of IAPs with their functional partners, such as caspases, should restore the cancer cell's apoptotic response to proapoptotic stimuli [24, 25]. RA-FLSs are resistant to apoptosis and this resistance and the increased proliferation of FLSs might contribute to the pathogenesis of RA [8, 9]. In a previous paper, we demonstrated, for the first time, that FLSs are sensitive to Smac mimetics [21]; here we evaluated the effects of more lipophilic, cell-permeable Smac127. Indeed, the trifluoromethyl substituent in Smac127 increased its lipophilicity and facilitated its permeation through cell membranes. Casnici et al. demonstrated that “in vitro” RA studies performed in the presence of SF in the culture medium recreate the physiopathological microenvironment of RA [22], because the composition of SF in RA pathology is very complex and strongly influences the microenvironment of joints. Furthermore, SF may contain many antiapoptotic factors that could reduce cells apoptosis. Thus, it was important to evaluate how Smac127 acted on RA-FLSs cultured in the presence of SF in the culture medium. To this purpose, we used a pool of SF from RA patients to minimize the variability in responses among the different individual SF samples. In this study we showed that Smac127 induced a significant inhibition of FLSs growth and induced apoptosis in RA-FLSs cultured in tissue medium or in SF (Figures 2 and 3). The percentage of apoptosis in cells cultured in SF was lower than in cells cultured in tissue medium alone, probably because of the presence in the SF of antiapoptotic factors. In our previous study [21] we showed that resistance to apoptosis in RA-FLSs depends on upregulation of IAPs. Indeed, Figure 4 shows that our molecule significantly inhibits the expression of IAP1, IAP2, and XIAP in RA-FLSs cultured in presence of both tissue medium and medium added with SF. We propose that administration of Smac127 could lead to rapid induction and proteasomal degradation of cellular IAPs and to the inhibition of canonical NF-*κ*B pathway [17], leading to upregulation of other survival proteins including Bcl-2 and Bcl-xL. [26, 27]. Smac127 enhanced the autoubiquitination of cIAPs, leading to their rapid destruction and thus allowing the activation of caspases inhibited by IAPs, when RA-FLSs were cultured either in tissue medium (Figure 5(a)) or in SF (Figure 5(b)). Probably, Smac127 disrupts IAP interaction with caspases or decreases intracellular levels of IAPs with distinct approaches, such as autoubiquitination-induced degradation, damaging the translation of IAP proteins, or inhibiting the transcription of IAPs. When cIAP1/2 are eliminated by IAP antagonists, a brief activation of the classical pathway NF-*κ*B occurs, presumably due to transient scaffolding from autoubiquitination, resulting in a short surge of TNF alpha expression, although TNF-independent apoptosis has been reported [24, 28, 29].

The overgrowth of FLSs results in the formation of typical RA pannus, which erodes surrounding bone [23], and in the production of proinflammatory cytokines, released by infiltrating mononuclear cells and lining synoviocytes, thus promoting and sustaining inflammation by both autocrine and paracrine pathways [29, 30], and the persistent imbalance between pro- and anti-inflammatory mechanisms leads to chronic inflammation and subsequent joint destruction.

IAPs influence the production of multiple inflammatory mediators, which are not only inhibitors of apoptosis, and here we demonstrated that Smac127 was able to inhibit IL-15 production in all tissue culture conditions. IL-15 is a potent proinflammatory cytokine, constitutively expressed on the surface of RA-FLSs, that plays a role in the pathogenesis of RA.

The blockade of IL-15 secretion by Smac127 is an important goal because it breaks the proinflammatory loop and decreases the resistance to apoptosis of FLSs [31]. The levels of IL-6 were very high in RA-FLSs cultured in the presence of SF, which reproduce the microenvironment within the inflamed joints, and this is consistent with the hypothesis of the presence of several prosurvival and proliferative factors, secreted by cells located within the joint [32, 33]. Interestingly, we found that Smac127 also inhibited IL-6 production when RA-FLSs were cultured both in tissue medium and in the presence of SF.

The intense synovial cell hyperplasia and proliferation are associated with a compensatory anti-inflammatory response characterized by the production of soluble TNF receptors, transforming growth factor (TGF-*β*), IL-1 receptor antagonist (IL-1RA), and IL-10. Particularly, levels of IL-10 are elevated in serum and SF of patients with RA and biologically significant quantities of functionally active IL-10 are released in the suspensions of rheumatoid synovial cell cultures [34–36]. Our results demonstrate that Smac127 significantly increased the production of IL-10 (Figure 6). This result is interesting because this cytokine has a dual anti-inflammatory activity, as, on the one hand, it decreases the secretion of proinflammatory cytokines IL-15 and IL-6 and on the other hand it stimulates its own secretion in RA-FLSs, particularly when cultured in SF. Here we did not investigate whether the stimulation of IL-10 secretion by Smac127 was responsible of the downregulation of IL-15 and IL-6 or if such downregulation is a direct effect of Smac127. The synergistic interactions among the different cytokines in the inflamed joint enhance the production of MMPs and subsequent joint destruction. MMPs determine the timing, the amplitude, and the combination of molecular signals that are released within a tissue, and crucially influence the availability of cell death or cell protective cues in the microenvironment, thereby affecting the outcome of an inflammatory challenge [37]. Since the regulation of MMPs becomes aberrant in immune cells in many human inflammatory and autoimmune diseases [37, 38], we also evaluated the effect of Smac127 on MMP-1 production. We observed that it reduced MMP-1 levels (Figure 6) and, interestingly, this inhibition was maintained even in RA-FLSs exposed to SF, where MMP1 levels were very high.

Other authors have shown that IAP antagonists may induce high turnover osteoporosis characterized by enhanced osteoclast and osteoblast activities in mice and may increase the risk of tumour growth and metastasis in the bone by stabilizing NF-*κ*B inducing kinase (NIK) and activating the alternative NF-*κ*B pathway in osteoclasts [39]. Therefore, we sought to find whether the RANKL/OPG pathway was altered by Smac127, both in cells cultured in tissue medium and in the presence of SF. As expected, RANKL is upregulated in cells cultured in the presence of SF, but we found no statistically significant difference in RANKL secretion after the addition of Smac127. Similarly, this compound was not able to influence the OPG production.

In conclusion, Smac127 demonstrated to be effective in inducing apoptosis in RA-FLSs which are characterized by resistance to apoptosis and are responsible for the erosive pannus typical of RA. We also demonstrated that Smac127 has a beneficial regulatory effect on the expression of proinflammatory cytokines and downregulates the production of MMPs. We did not confirm previous observations regarding a possible side effect on bone due to upregulation of RANKL and downregulation of OPG. Overall, these observations suggest a potential use of Smac mimetic compounds in general, and Smac127 in particular, for the control of inflammation and disease progression in RA.
